# The Cellular Immune Response of the Pea Aphid to Foreign Intrusion and Symbiotic Challenge

**DOI:** 10.1371/journal.pone.0042114

**Published:** 2012-07-27

**Authors:** Antonin Schmitz, Caroline Anselme, Marc Ravallec, Christian Rebuf, Jean-Christophe Simon, Jean-Luc Gatti, Marylène Poirié

**Affiliations:** 1 Institut National de la Recherche Agronomique (INRA), Unité Mixte de Recherches 1355 “Institut Sophia Agrobiotech” (ISA), Sophia Antipolis, France; 2 Centre National de la Recherche Scientifique (CNRS), Unité Mixte de Recherches 7254, Sophia Antipolis, France; 3 Université Nice Sophia Antipolis, Nice, France; 4 Institut National de la Recherche Agronomique (INRA) - Université Montpellier 2, Unité Mixte de Recherches 1333 “Diversité, Génomes et Interactions Microorganismes-Insectes”, Montpellier, France; 5 Institut National de la Recherche Agronomique (INRA), UMR 1349, Institut de Génétique, Environnement et Protection des Plantes, Le Rheu, France; Kansas State University, United States of America

## Abstract

Recent studies suggest that the pea aphid (*Acyrthosiphon pisum*) has low immune defenses. However, its immune components are largely undescribed, and notably, extensive characterization of circulating cells has been missing. Here, we report characterization of five cell categories in hemolymph of adults of the LL01 pea aphid clone, devoid of secondary symbionts (SS): prohemocytes, plasmatocytes, granulocytes, spherulocytes and wax cells. Circulating lipid-filed wax cells are rare; they otherwise localize at the basis of the cornicles. Spherulocytes, that are likely sub-cuticular sessile cells, are involved in the coagulation process. Prohemocytes have features of precursor cells. Plasmatocytes and granulocytes, the only adherent cells, can form a layer *in vivo* around inserted foreign objects and phagocytize latex beads or *Escherichia coli* bacteria injected into aphid hemolymph. Using digital image analysis, we estimated that the hemolymph from one LL01 aphid contains about 600 adherent cells, 35% being granulocytes. Among aphid YR2 lines differing only in their SS content, similar results to LL01 were observed for YR2-Amp (without SS) and YR2-Ss (with *Serratia symbiotica*), while YR2-Hd (with *Hamiltonella defensa*) and YR2(Ri) (with *Regiella insecticola*) had strikingly lower adherent hemocyte numbers and granulocyte proportions. The effect of the presence of SS on *A. pisum* cellular immunity is thus symbiont-dependent. Interestingly, *Buchnera aphidicola* (the aphid primary symbiont) and all SS, whether naturally present, released during hemolymph collection, or artificially injected, were internalized by adherent hemocytes. Inside hemocytes, SS were observed in phagocytic vesicles, most often in phagolysosomes. Our results thus raise the question whether aphid symbionts in hemolymph are taken up and destroyed by hemocytes, or actively promote their own internalization, for instance as a way of being transmitted to the next generation. Altogether, we demonstrate here a strong interaction between aphid symbionts and immune cells, depending upon the symbiont, highlighting the link between immunity and symbiosis.

## Introduction

Insect defense against pathogens relies on innate immune mechanisms that have mainly been characterized in Diptera and Lepidoptera [1]–[3], and show substantial conservation in Hymenoptera and Coleoptera [4], [5]. In comparison, the immunity of hemimetabolous insects has been fewly investigated, although they comprise economically important species. The recent annotation of the genome of the pea aphid *Acyrthosiphon pisum* [6] suggests that many components central to immune functions in other insects are missing. These include antimicrobial peptides (AMPs) such as defensins, PeptidoGlycan Recognition Proteins (PGRPs), some of the cellular Pattern Recognition Receptors (PRRs) and several components of the Imd pathway [7]. Besides, all experiments failed to identify significant changes in expression of immunity genes in response to wounding, stress or pathogen challenges [7], [8]. However, genomic approaches are only predictive and, for instance, an extensive description of the nature and defense functions of aphid hemocytes remained to be provided.

Another interest of aphids is their obligate association with a primary symbiont, *Buchnera aphidicola (Ba)*, and the large number of facultative secondary symbionts they can host (e.g. *Serratia symbiotica (Ss)*, *Hamiltonella defense (Hd)*, *Regiella insecticola (Ri)*). Indeed, it has recently been demonstrated that symbionts can significantly influence the host immune response [9]–[11]. For instance, the presence of the alpha proteobacteria *Wolbachia* is associated with increased susceptibility to parasitoid infection in *Drosophila simulans* [12] or with alteration of immune components in the woodlouse *Armadillidium vulgare* [13], and it mediates protection against RNA virus infection in *D. melanogaster* [14]. The maintenance of symbiosis with regard to the host immunity has also been investigated. Symbionts seem to be detected by the host immune system [15], [16]; they can be observed inside host hemocytes [13], [16], possibly as a way of protecting themselves from host defenses [17]. In addition, the host immune system can control the division of symbionts and their localization, as demonstrated recently [18].

In the pea aphid, some secondary symbionts are involved in host resistance to fungal pathogens [19] and parasitoid wasps [20], through mechanisms yet only partly identified. In addition, while primary symbionts are usually confined to the cytoplasm of specialized cells, the bacteriocytes, secondary symbionts are also observed free in the hemolymph [21], [22], raising the question of how the immune system responds to the presence of symbionts and whether symbionts may somehow affect aphid immunity. In the predicted absence of strong humoral immunity, aphid immune cells are the main components with which symbionts may interact. However, there are few available descriptions of these cells, and they only rely on light microscopy observations (see [23] as an example). The first data on *A. pisum* were reported only recently, with the description of three morphologically distinct types of hemocytes: prohemocytes that may correspond to stem cells, granulocytes that phagocytize bacteria, and oenocytoids that exhibit melanotic activity [24].

Here, we deepen the knowledge of *A. pisum* hemocytes by providing extensive morphological and functional characterization, based on light and electron microscopy. We describe a new cell category (the plasmatocytes) and decipher the role of the different hemocytes in phagocytosis, coagulation, adhesion and encapsulation. We also show that, besides being able to engulf foreign bodies, plasmatocytes and granulocytes actively phagocytize primary and secondary symbionts when present in the hemolymph. Finally, we report a lower number of adherent cells and a smaller granulocyte/plasmatocyte proportion in presence of some, but not all, secondary symbionts. Aphid symbionts thus strongly and diversely interact with the cellular immunity of their host, a finding that opens new avenues of research to question the link between symbiosis and immunity.

## Results

### Hemocyte characterization

Hemocyte characterization was primarily performed on a clone devoid of secondary symbionts (LL01). Five main cell types were defined using several morphological and functional criteria (e.g. size, nucleus/cytoplasm ratio (N/C), morphology, adhesion properties, staining properties). These cells categories were later observed in all the aphid lines tested.

**Figure 1:**
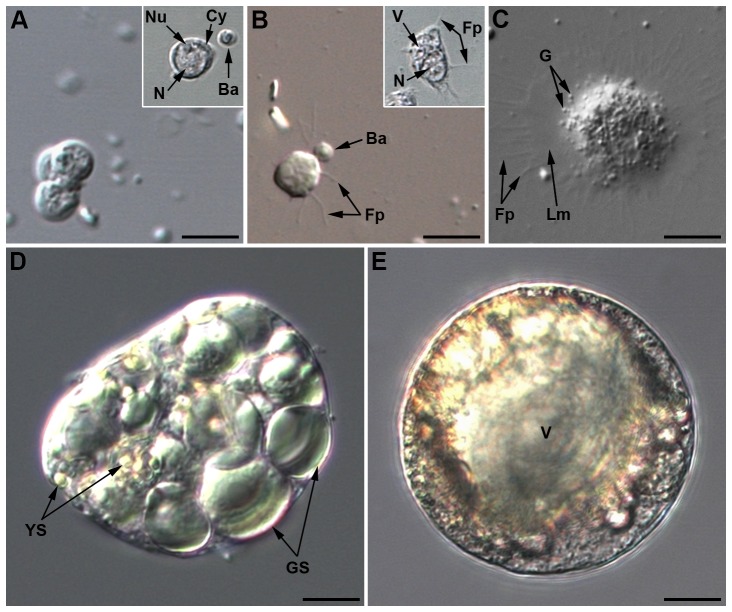
Light microscopy pictures of *Acyrthosiphon pisum* hemocytes (LL01 clone). (A) Three prohemocytes in cluster. Inset: phase contrast showing the large central nucleus and the nucleolus (*Nu*). Ba: *B. aphidicola*. (B) Plasmatocyte beginning to adhere, with filopodia (*Fp*) extension. Inset: phase contrast showing large cytoplasmic vacuolar formation. (C) Adherent granulocyte containing cytoplasmic granules (*G*) and filopodia (*Fp*) extending from a lamellipodium (*Lm*). (D) Spherulocyte with its large colored globular inclusions, small yellow spherules *(YS)* and large green spherules *(GS)*. (E) Wax cell showing a large central vacuole *(V)* and colored globular inclusions that differ from those of spherulocytes. Same magnification for all micrographs; scale bar: 10 µm.

**Figure 2:**
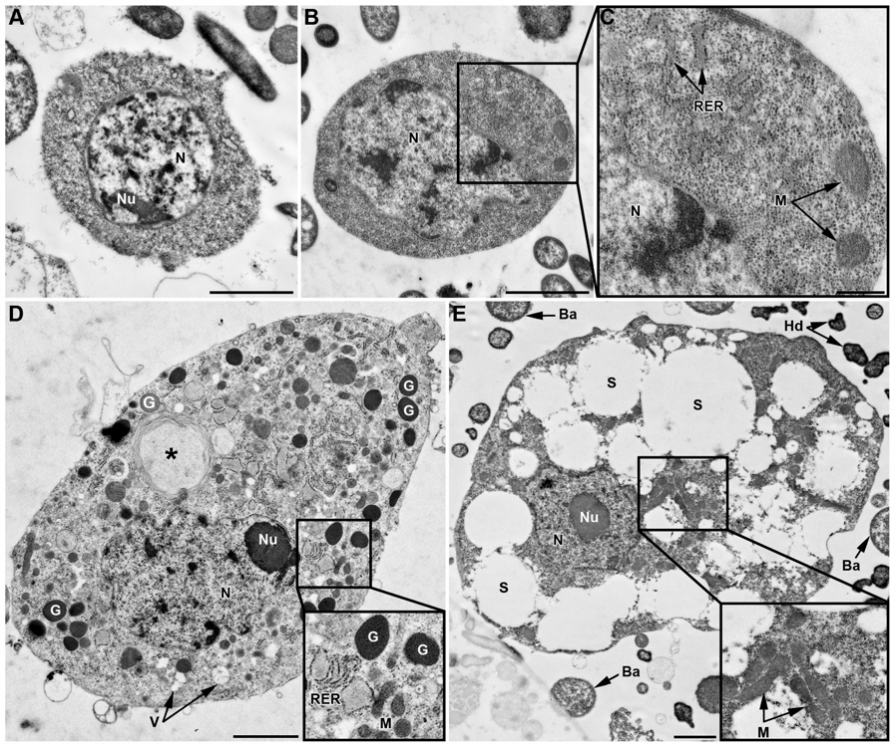
TEM characterization of hemocytes. Cells from the LL01 clone (A–D) and the YR2-Hd line (E). (A) Prohemocyte characterized by a round nucleus (*N*), a high N/C ratio, and homogenous cytoplasm devoid of apparent organelles. (B) Plasmatocyte with homogeneous cytoplasm, a lobulated nucleus, a high N/C ratio and some cytoplasmic organelles clearly visible in box (C): Rough Endoplasmic Reticulum (*RER*), Mitochondria (*M*). (D) Granulocyte with a lobulated nucleus, a low N/C ratio, and granules *(G)*. The cytoplasm contains numerous organelles (see box). A phagosome is observed that contains a large foreign particle (asterisk). (E) YR2-Hd spherulocyte with a round nucleus and a low N/C ratio. The large volume of cytoplasm is filled with spherules (*S*) of different sizes, and numerous mitochondria are found in a small region (enlarged box). *Ba*: *B. aphidicola*; *Hd*: *H. defensa*. Scale bar: 2 µm (A, B, D, E) and 0.5 µm (C).

*Prohemocytes* are the smallest cells in hemolymph, often observed in clusters of three or more cells (Fig. 1A). Their diameter ranges from 5 to 8 µm; they have a spherical shape and a very high N/C ratio (Fig. 1A inset and Fig. 2A). The central spherical nucleus occupies most of the cell and is surrounded by a limited layer of basophilic cytoplasm (May-Grünwald Giemsa (MGG) staining; not shown). A small nucleolus can be observed by light microscopy (Fig. 1A inset) and transmission electron microcopy (TEM) (Fig. 2A). TEM observations showed a well-defined nuclear envelope and an homogenous cytoplasm filled with very small dense particles and also containing a few vesicles (Fig. 2A). Organelles such as mitochondria, Golgi apparatus or endoplasmic reticulum were barely distinguishable. Prohemocytes never showed cytoplasmic extensions; they were never retrieved on adherent hemocyte preparations (named thereafter AHPs; see material and methods), nor adhering onto foreign material, and were never observed phagocytizing injected particles.

**Figure 3:**
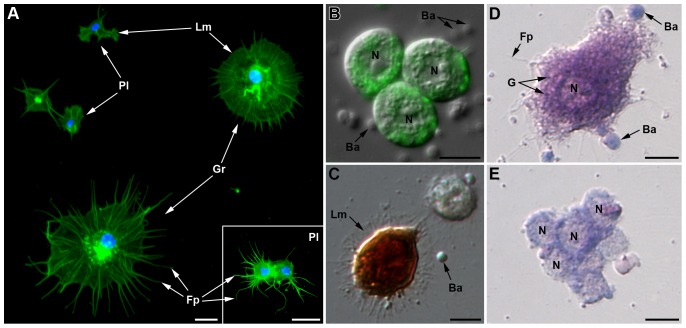
Histological characterization of adherent hemocytes (LL01 clone). (A) Fluorescent micrograph showing AHP after F-actin (green) and nucleus (blue) staining. Plasmatocytes (*Pl*) are the smaller cells; they display two adhesion profiles (i) lamellipodia (*Lm*) extension without apparent filopodia or (ii) filopodia extension (*Fp*) exclusively (inset). Granulocytes (*Gr*) have spread more than four times their original size; they display lamellipodia with radiates filopodia. (B) Merger of DIC and fluorescent micrographs showing three clumped ROS-producing granulocytes (green fluorescence of Rhodamine 123). (C) Intracellular PO staining on AHP using Dopamine hydrochloride showing a PO-positive hemocyte (brown staining). (D–E) Adherent hemocytes stained with May Grünwald Giemsa. (D) Granulocyte with an eosinophilic cytoplasm (pink staining) that contains basophilic granules (blue staining). (E) Clumped plasmatocytes with a limited layer of basophilic cytoplasm. *Ba*: *B. aphidicola*. Scale bars: 10 µm.

*Plasmatocytes* are small round or occasionally spindle-shaped cells, of 8 to 10 µm in diameter, with a high N/C ratio (Fig. 1B, 2B–C). Their eccentric nucleus is round to lobulated, with a well-developed nucleolus. The basophilic cytoplasm (blue reaction with MGG, Fig. 3E) is homogenous, but cytoplasmic vacuoles were sometimes observed by phase contrast microscopy (Fig. 1B, inset). By light microscopy, plasmatocytes differed from prohemocytes by their extended filopodia (Fig. 1B). Under TEM observation (Fig. 2B–C), the nucleus had a lobulated shape and the cytoplasm, almost devoid of vesicles, contained clearly visible cytoplasmic organelles (mitochondria, free ribosomes, rough endoplasmic reticulum (RER)). Plasmatocytes have adhesive properties: they attach to glass and can form clusters when entering in contact with each other (inset of Fig. 3A, Fig. 3E). By F-actin staining on AHPs, two types of adhesion profile were observed (Fig. 3A): filopodia extension exclusively (inset of Fig. 3A), or lamellipodium extension without apparent filopodia (Fig. 3A). Plasmatocytes were also weakly positive for the presence of ROS (not shown).

*Granulocytes* are usually spherical or ovoid, with a 10 to 20 µm diameter. They have a low N/C ratio and an eccentric, generally lobulated nucleus, with a prominent nucleolus. Under light microscopy, vacuoles and filamentous processes are observed as well as granules that could be released by the cell (Fig. 1C). TEM observation (Fig. 2D) showed numerous cytoplasmic organelles (mitochondria, Golgi apparatus, free ribosomes, and RER), suggesting they are highly active cells. In addition, large cytoplasmic granules (up to 1 µm in diameter), filled with more or less electron dense material, could be observed scattered at the cell periphery. Granules' variation in size and electron density may be due to a maturation process. In addition to granules, cytoplasmic clear membrane vesicles, as well as phagosome-like structures surrounding ingested particles were observed. Granulocytes readily adhered to glass coverslips and were able to spread more than four times their original size one hour after hemolymph collection (Fig. 3A). Spreading occurred in a fan-like manner with star-like filopodia extending from lamellipodia. Most granulocytes spread symmetrically but some spread asymmetrically along one axis (Fig. 3A, 3D). Granulocytes often clumped and spread when they entered in contact with each other (Fig. 3B). The eosinophilic cytoplasm was more or less filled with basophilic granular inclusions (MGG staining, Fig. 3D), suggesting that granulocytes might degranulate in the course of the experiment. Granulocytes were strongly positive for the presence of ROS (Fig. 3B). About 2% (2.39±1%) of adherent hemocytes on AHPs displayed a strong intracellular PO staining (Fig. 3C). Since these cells had the same size and shape, as well as similar lamellipodia/filopodia extensions as granulocytes, they were considered as such.

*Spherulocytes* were observed both in freshly collected hemolymph (Fig. 1D), and adherent under the cuticle (not shown), suggesting they might be sessile cells that were released upon collection. They are very large (from 20 to 100 µm in diameter), highly polymorphic, with a spherical to undefined shape; they have a low N/C ratio (Fig. 1D). Their round eccentric nucleus contains a well-developed central nucleolus (intense blue coloration with methyl blue, Fig. S1). Highly colored prominent vesicles (globules or spherules) are found in their cytoplasm, with large green spherules (>5 µm in diameter) and small yellow spherules (about 1–2 µm in diameter) (Fig. 1D). We observed that the color of the spherules in different clones matched that of the aphid body (Table 1), as previously reported [25].

**Table 1:** Origin and characteristics of the aphid lines.

Lines	Color	Plant origin	Location	Collection date	SS composition (donor line)
LL01	Green	Alfalfa	Lusignan (France)	1987	none
YR2(Ri)	Pink	Clover	York (England)	2002	*R. insecticola*
YR2-Amp*	Pink	-	-	-	none
YR2-Hd*	Pink	-	-	-	*H. defensa* (L1-22)
YR2-Ss*	Green	-	-	-	*S. symbiotica* (P136)

* : artificial lines.

Spherulocytes are fragile cells that broke easily during collection, leaving free nuclei in suspension in the medium (Fig. S1). Accordingly, within 30 min following collection, numerous spherules were floating at the surface of the collected hemolymph. This likely explains why we rarely observed these cells using TEM. The only observation (Fig. 2E, from the clone YR2-Hd) showed many empty vacuoles that matched in size and location with the two types of spherules, as well as numerous mitochondria concentrated in a restricted region of the cytoplasm. Spherulocytes attached very poorly to glass and did not show filamentous processes.

**Figure 4:**
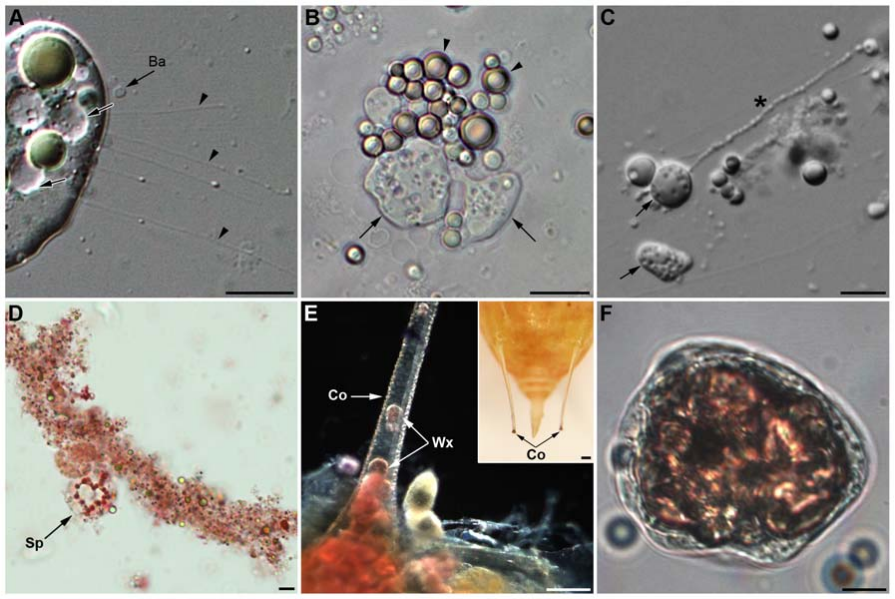
Functions of spherulocytes and wax cells. (A–C) Early alterations of spherulocytes after hemolymph collection: (A) loss of spherules and fibrils' formation (arrowhead). *Ba*: *B. aphidicola*. (B) Unstable cytoplasmic blebs (arrow) derive from spherulocytes while spherules remain intact (arrowhead). (C) From blebs (arrow), long stable strands like strings of pearls extend (asterisk; see also movie 1). (D) Low magnification of a large coagulum stained by neutral red showing a granular aspect and the presence of an intact spherulocyte (*Sp*). (E–F) Wax cells (*Wx*) are localized at the base and inside the cornicles (*Co*), secretory appendices localized at the posterior end of aphids' bodies (inset in (E)). (E) Red lipid staining showing large accumulation of lipid-containing cells at the base of the cornicle. (F) Wax cell containing a large neutral lipidic inclusion (pink staining) that almost fills entirely the cytoplasm. Scale bars: 15 µm (A), 10 µm (B, C, D, F) and 100 µm (E and inset in (E)).

A main function of spherulocytes is their involvement in the coagulation process that occurred rapidly following hemolymph collection (Fig. 4A–D, movie S1). Spherulocytes underwent a succession of modification steps that began with a loss of spherules and formation of fibrils (Fig. 4A), followed by rapid expansion of the cytoplasm, with the formation of a beam-like protuberance from cytoplasmic swellings and blebs (Fig. 4B) that rapidly changed into long strings of pearls (Fig. 4C). The process more or less extended and it ended with the organization of a granular coagulum into a delicate meshwork of fibrils (Fig. 4D). The presence of numerous spherules and the high number of mitochondria suggest that spherulocytes may also be involved in energy storage.

*Wax cells* were sometimes observed in hemolymph preparations (Fig. 1E) but they were mainly localized at the base and in the canal of the aphid cornicles (Fig. 4E). These spherical to ovoid cells have a 40–50 µm diameter and a low N/C ratio. They have a very low density, floating at the surface of the collected hemolymph, and their cytoplasm is more or less filled with large neutral lipid inclusions (Nile blue sulfate staining, Fig. 4F), in agreement with their involvement in wax production and lipid storage [26]. Wax cells never showed cytoplasmic extensions nor adhesive properties. They were never observed in TEM preparations, likely because they were lost during the centrifugation steps. Because of their restricted localization and putative functions, they were not considered as true circulating hemocytes.

### Adherent hemocyte number and granulocyte proportion in different aphid lines

**Figure 5:**
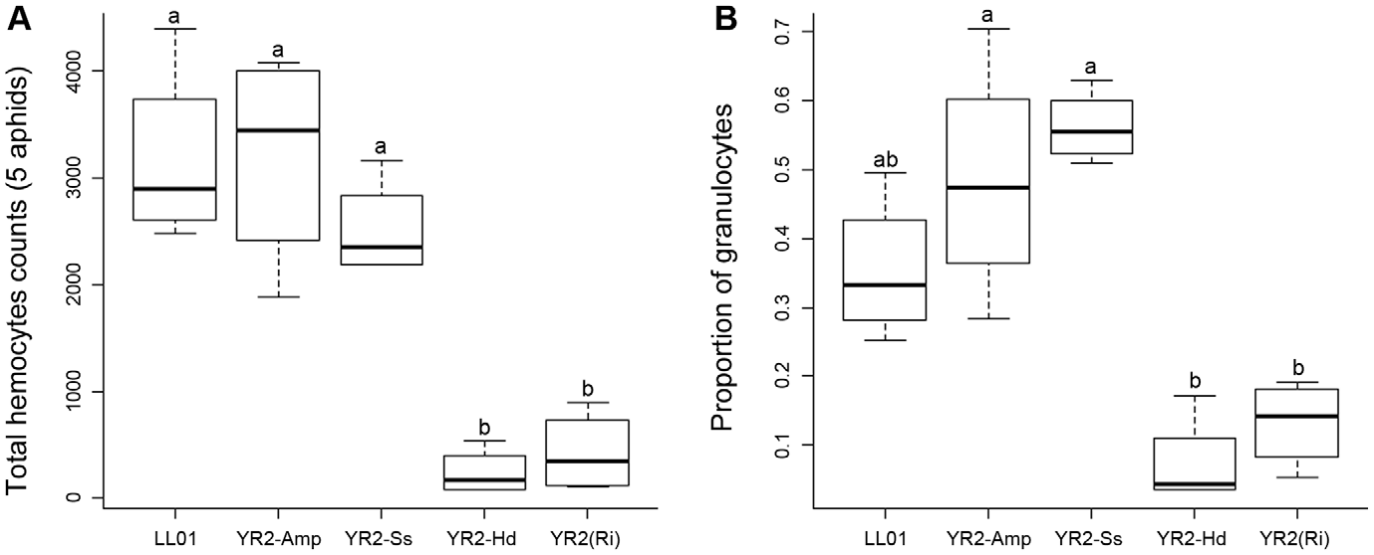
Total hemocyte counts (THC) and granulocyte proportions from AHPs of the different lines. Box-plot representations of the THC (A) and the proportion of granulocytes (B), estimated on F-actin stained AHPs (pools of 5 adults per AHP), for aphid lines listed in Table 1. Box-plots with the same letter have means that are not significantly different (TukeyHSD, alpha = 5e-03, n = 4).

Due to the hemolymph contamination and spherulocytes' leakage observed with all collection methods, the respective abundance of hemocyte categories in aphid hemolymph could not be accurately determined. However, spread cells were readily recognizable after F-actin staining by their filamentous processes (Fig. 3A). We then developed an image recognition method based on AHPs (Methods S1, Fig. S2) to estimate the number of adherent hemocytes and the proportion of granulocytes and plasmatocytes in aphid lines (Fig. 5). The total adherent hemocyte count (THC) (Fig. 5A) was about 3171±634 for five pooled LL01 aphids (634±170 cells per individual), but it strongly differed among aphid lines (ANOVA, p = 7.0e-06). No significant difference was found between LL01 and YR2-Amp, the lines devoid of secondary symbionts (SS) (TukeyHSD, p = 1), and between YR2-Amp and YR2-Ss (TukeyHSD, p = 0.58). In contrast, THCs from YR2(Ri) and YR2-Hd were on average 7.6 times and 13.4 times lower than that of YR2-Amp, respectively (TukeyHSD, p = 2.0e-04 and p = 1.0e-04). The proportion of granulocytes differed among aphid lines similarly to the THC (Fig. 5B; ANOVA, p-value = 1.5e-05), being on average 3.7 times and 6.6 times lower in YR2(Ri) and YR2-Hd compared to YR2-Amp, respectively (TukeyHSD, p = 1.6e-03 and p = 3.5e-04). Contrastingly, there was no difference between the lines devoid of SS, LL01 and YR2-Amp (TukeyHSD, p = 0.41), or between YR2-Amp and YR2-Ss (TukeyHSD, p = 0.81). In lines devoid of SS, granulocytes (including PO-positive hemocytes) represented 35% (LL01) and 48% (YR2-Amp) of adherent hemocytes, others being plasmatocytes.

### Hemocyte adhesion *in vivo*

**Figure 6:**
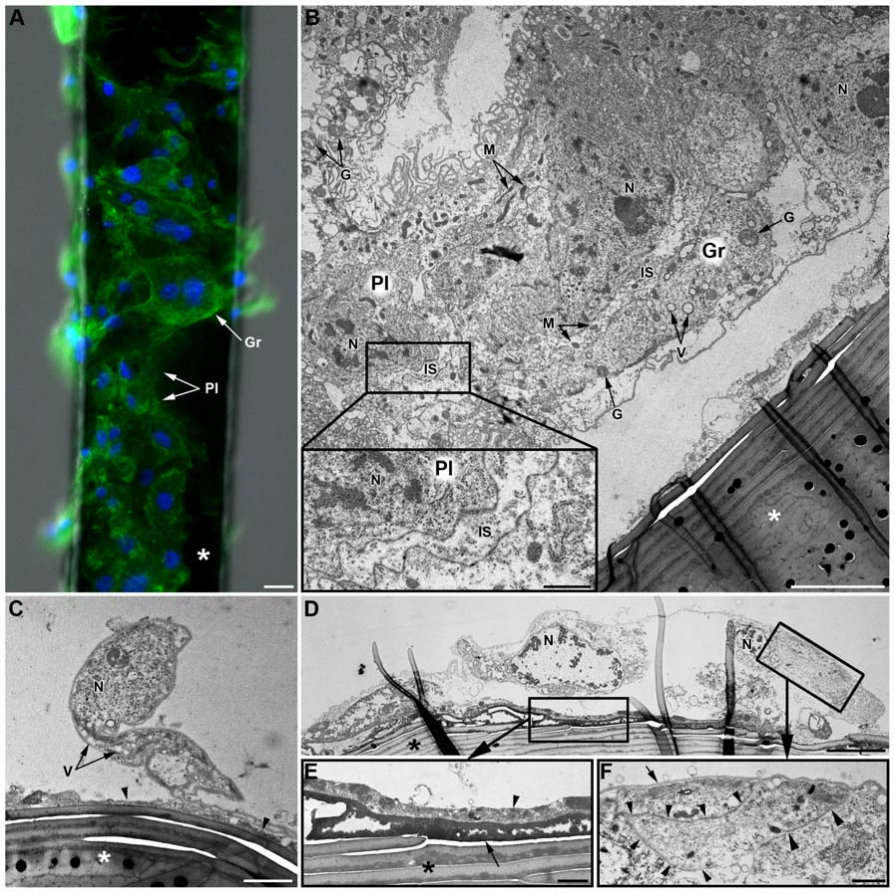
*In vivo* hemocyte adhesion assay (LL01 clone). (A) Observations of a brush horsehair (asterisk) seven days after insertion in the hemocoele of an LL01 aphid: both plasmatocytes (*Pl*) and granulocytes (*Gr*) are observed (F-actin (green) and nuclei (blue) staining). Scale bar: 10 µm. (B–F) TEM micrographs of the “encapsulation” process. (B) Part of a multilayered hemocytic capsule containing both plasmatocytes (Pl) and granulocytes (*Gr*). The capsule is partly detached from the brush hair (asterisk). Intercellular spaces (*IS*) between hemocytes are relatively large (see box magnified bottom left) and they often contain some cytoplasmic organelles (mitochondria, granules…). Scale bar: 5 µm; box: 1 µm. (C) Plasmatocyte partly adhering to the brush hair (asterisk). An electron-dense matrix of granular aspect is observed at the interface between the hair and the hemocyte (arrowhead). Scale bar: 2 µm. (D) Granulocyte largely spread onto the hair. Two characteristic layers are observed at the interface between the horsehair and the hemocyte (box magnified in (E)). Scale bar: 5 µm. (E) The internal layer is highly electron-dense, compact and homogenous (arrow). The external layer is heterogeneous and composed of cell debris and coagulated hemolymph (arrowhead). Scale bar: 1 µm. This granulocyte is in tight contact with another “capsule”-forming hemocyte (box magnified in (F); arrowheads). An electron-dense thin layer covers the surface of the cell (arrow). Scale bar: 1 µm. *N*: Nucleus; *M*: Mitochondria; *G*: Granule; *V*: Vacuole.

Adhesion properties of hemocytes were tested *in vivo* by inserting brush horsehair fragments in LL01 aphids. These fragments were removed at different times and observed by microscopy. Adhesion of some hemocytes was already observed 24 h post-insertion, and their number increased 48 h post-insertion (data not shown). One week after insertion, hairs were covered by adherent hemocytes forming a more or less extended cell monolayer, but which never happened to be complete (Fig. 6A ; 3D reconstruction in movie S2). To question if this was due to the large size of the hairs, and whether hemocytes could adhere to non-organic material, the experiment was repeated with smaller epoxy resin and glass capillary fragments. The same incomplete hemocyte layer was observed, together with melanin deposits (data not shown) that could not be observed on the black brush hairs. Interestingly, most of the inserted objects covered with hemocytes were found attached to aphid tissues such as the fat body, the trachea or the digestive tract. At the ultrastructural level, the cell monolayer was composed of both plasmatocytes and granulocytes, distinguishable by their morphology (size, N/C ratio) and the presence of granules (Fig. 6). Hemocytes occurred as either isolated cells (Fig. 6C–D) or as multicellular aggregates containing both cell categories, without any apparent organization (Fig. 6B). At places, the membranes of neighbor hemocytes were in tight contact, with no intercellular space (Fig. 6F), while in large cell aggregates intercellular spaces up to 2 µm were observed (Fig. 6B). Those spaces contained cellular debris (mitochondria, granules…) possibly originating from cell lysis. An electron dense layer was always observed at the interface between hemocytes and horsehairs (Fig. 6C–E), as well as on the surface of the horsehair even in areas devoid of hemocytes. This layer had a granular aspect when observed at high magnification (thickness about 30 nm per granule) and its appearance varied from a granular deposit of low electron density (grey color, Fig. 6C) to a compact, less granular, electron dense matrix (black, Fig. 6D). A second layer of material was sometimes observed between this dense matrix and the hemocytes' membrane (Fig. 6E) that had a slightly granular aspect and contained cellular organelles (e.g. mitochondria). Besides, a thin layer of electron-dense material was observed at the surface of hemocytes facing the hemocoele cavity (Fig. 6F).

### Phagocytosis of latex beads and *E. coli* bacteria

The hemocyte capacity to internalize foreign particles was tested by injecting latex beads or fluorescent *E. coli* DH5-alpha bacteria inside the body cavity of LL01 aphids. Similar experiments were performed with YR2-Amp aphids, with similar results (data not shown).

**Figure 7:**
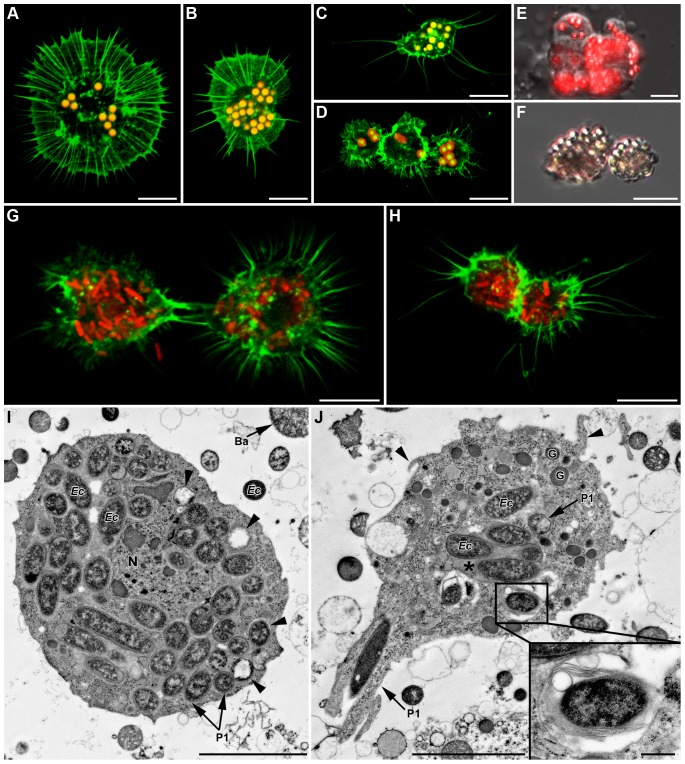
*In vivo* phagocytosis of fluorescent latex beads and fluorescent *E. coli* 24 h post-injection in LL01 aphids. (A–D) Confocal images of both granulocytes (A and B) and plasmatocytes (C and D) having ingested numerous latex beads (yellow-red fluorescence). (E) Merger of DIC and fluorescent micrographs showing an aggregate of hemocytes actively phagocytizing beads (red fluorescence). (F) Melanization of ingested latex beads (brown staining). (G–H) Granulocytes (in G) and plasmatocytes (in H) containing numerous red fluorescent bacteria (F-actin in green); Scale bar: 10 µm (A–H). (I–J) TEM micrographs showing uptake and degradation of ingested bacteria. (I) *E. coli* bacteria inside the cytoplasm of a granulocyte (arrowheads: bacteria at different degrees of degradation). (J) Granulocyte phagocytizing a bacterium in a zipper-like manner (*P1* arrow). Phagolysosome-like structures are seen that contain a single bacterium (magnified in box) or several bacteria in an electron-dense matrix (asterisk). See also filopodial extensions (arrowhead). Scale bar: 5 µm (I–J) and 0.5 µm (magnifications box in (J)).

The cellular uptake of injected particles was observed by fluorescent microscopy, and intracellular localization was confirmed using confocal microscopy and TEM. Prohemocytes, spherulocytes and wax cells did not contain any latex bead or fluorescent bacteria whatever the time post-injection (data not shown), thus being classified as non-phagocytic cells. In contrast, both granulocytes and plasmatocytes mounted a strong and rapid phagocytic response (as soon as 2 h post-injection, data not shown) toward abiotic (latex beads, Fig. 7A–F) and biotic (*E. coli*, Fig. 7G–J) particles. At all observation times (2 h, 5 h and 24 h), the cytoplasm of many adherent cells was filled with latex beads or bacteria, granulocytes being able to take up more particles than plasmatocytes due to their bigger size. We estimated that 27% and 35% of adherent hemocytes had taken up three or more latex beads 5 h and 24 h post-injection, respectively. 3D projections generated from confocal optical sections demonstrated that beads and bacteria are indeed inside the cells, and that a single hemocyte could phagocytize more than 50 beads or bacteria (see movie S3 for latex beads engulfment in 3D projection). In addition, beads-containing hemocytes frequently formed aggregates (Fig. 7E) and some of them were totally melanized 24 h post injection (Fig. 7F). Adherent hemocytes containing numerous particles had a modified morphology, with less cytoplasmic extensions (lamellipodia and filopodia) and spreading ability. The latex beads inside hemocytes could not be visualized using TEM, likely because beads were lost during sample processing, but empty prints of the bead size were found in the cytoplasm (not shown). By contrast, many hemocytes from aphids injected with *E. coli* had their cytoplasm filled with bacteria (up to 45 bacteria observed by TEM on one cell slice), all tightly enclosed in different types of phagosomal structures (Fig. 7I–J). Phagosomes observed on TEM were classified on a morphological basis according to [27] (see also Figure legends). Some internalized bacteria showed variable degrees of degradation as judged by the loss of internal structure (Fig. 7I). Despite this intense phagocytosis, many bacteria remained free in the hemolymph 24 h post-injection.

### Phagocytosis of symbionts

#### Phagocytosis of secondary symbionts

**Figure 8:**
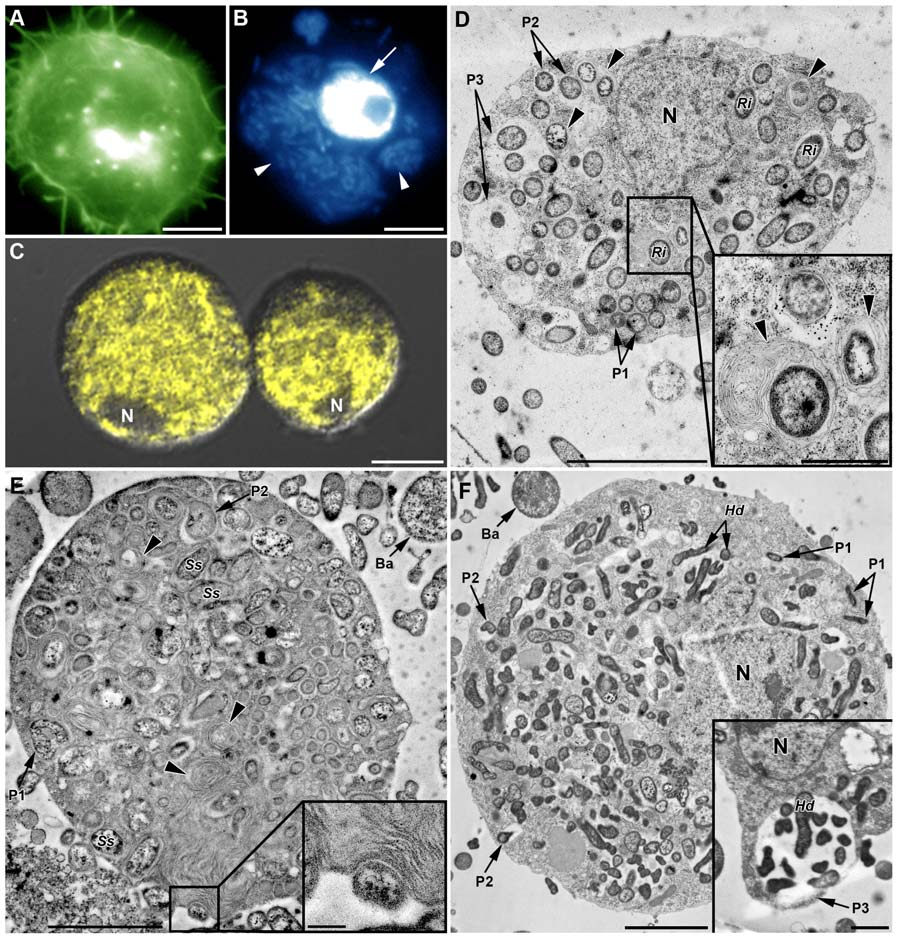
*In vivo* phagocytosis of symbionts by hemocytes from SS-containing YR2 lines. (A–C) Fluorescent micrographs of YR2(Ri) hemocytes. (A) F-actin (green) and (B) DNA staining (blue), of an adherent granulocyte. The cytoplasm is filled with *R. insecticola* secondary symbionts as shown by bacterial DNA staining (arrowheads in B; arrow: cell nucleus), and (C) specific FISH detection (yellow, false color; DIC and confocal micrographs overlay). Nucleus (*N*) location is detectable by the absence of yellow coloration. (D–F) TEM micrographs showing phagocytosis of secondary symbionts by hemocytes of three YR2 lines. (D) YR2(Ri) granulocyte containing numerous *R. insecticola* inside the cytoplasm. Different symbiont-containing phagosomal structures are observed (*P1*: zipper-like, *P2*: trigger-like, *P3*: macropinocytosis), as well as phagolysosomes containing symbionts and membranous material (arrowheads and box). (E) YR2-Ss line granulocyte with numerous *S. symbiotica* inside cytoplasmic membranous phagolysosomes (arrowheads). Insert box: detail of *S. symbiotica* engulfment by trigger-like phagocytosis. (F) YR2-Hd granulocyte containing *H. defensa* symbionts in the three types of phagosomes. Inset: plasmatocyte with a large phagosome structure (macropinocytosis-like) enclosing several symbionts and extracellular fluid. Scale bar: 5 µm (A–B), 10 µm (C), 5 µm (D), 2 µm (F) and 1 µm (magnifications boxes in (D) and (E)).

Surprisingly, observation of AHPs from YR2(Ri), YR2-Hd and YR2-Ss lines, whose hemolymph contains a high number of their respective secondary symbionts (SS), revealed the presence of SS inside the hemocytes. A high proportion of plasmatocytes and granulocytes had their cytoplasm full of bacteria, whose identity was confirmed using specific FISH (data shown for YR2(Ri), Fig. 8B–C). Occurrence of phagocytosis was confirmed by TEM, with up to 50 *R. insecticola* (YR2(Ri)), 100 *S. symbiotica* (YR2-Ss), and 180 *H. defensa* (YR2-Hd) observed on a single slice of one granulocyte of the corresponding line (Fig. 8D–F).

In YR2(Ri), phagolysosome-like structures were found that contained *Ri* bacteria, but the bacteria did not present visible signs of degradation. In YR2-Ss, numerous phagosomes with concentric multiple membranes were observed, some but not all containing *Ss* bacteria (Fig. 8E). The absence of *Ss* in part of these phagolysosome-like structures could possibly result from the complete degradation of the bacteria. Finally, in the YR2-Hd line, *Hd* symbionts were found in phagosomes but no phagolysosome-like structure could be observed.

**Figure 9:**
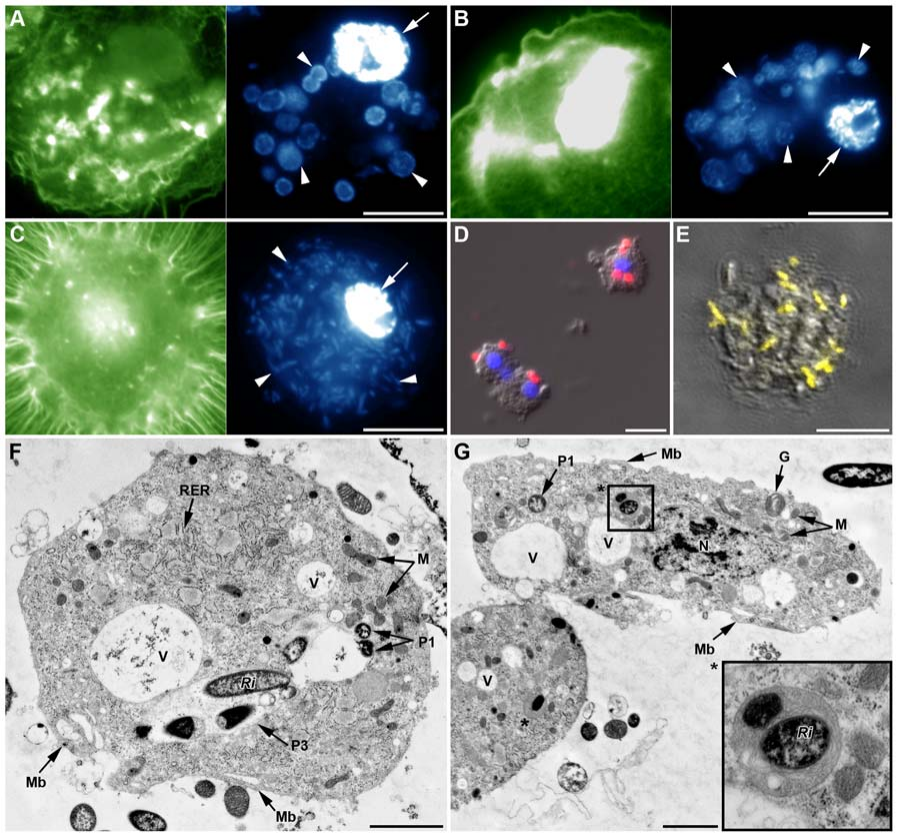
*In vivo* phagocytosis of primary symbionts and injected secondary symbionts in LL01 and YR2-Amp aphids (devoid of SS). (A–B) Fluorescent images of AHP from LL01 (A) and YR2-Amp (B) unchallenged lines. Hemocytes have taken up *B. aphidicola* (*Ba*) cells (arrowheads); F-actin in green, DNA in blue, arrows indicate cell nuclei. (C) Fluorescent images of AHP from the LL01 line, 24 h after injection of *R. insecticola* (*Ri*) secondary symbionts. *Ri* are observed in the cytoplasm of a granulocyte (arrowhead); F-actin in green, DNA in blue, arrows indicate cell nuclei. (D) Overlay of DIC and fluorescent images from AHP of unchallenged LL01 aphids, showing FISH detection of *Ba* cells (red fluorescence) in contact or within adherent hemocytes. Nuclei are counterstained with DAPI (blue fluorescence). (E) Overlay of DIC and confocal images showing FISH detection of *Ri* secondary symbionts (yellow fluorescence, false color) in a granulocyte of an LL01 aphid, 24 h post-injection. (F–G) TEM showing secondary symbionts *Ri* within LL01 hemocytes 24 h post-injection. Notice the high vacuolization (*V*: Vacuoles), the presence of membrane ruffles (*Mb*) and the phagosomes containing ingested *Ri*. (F) LL01 Plasmatocyte showing two zipper-like phagosomes containing individual bacteria (*P1*) and a macropinocytosis-like phagosome (*P3*) containing several symbionts, some in advanced state of degradation (high darkening). (G) LL01 granulocytes showing zipper-like phagosome (*P1*) and phagolysosomes containing symbionts (asterisk and box magnified). Scale bar: 10 µm (A–E), 1 µm (F) and 2 µm (G). *N*: Nucleus; *M*: Mitochondria; *RER*: Rough endoplasmic reticulum; *G*: Granule.

To determine if secondary symbionts freshly injected in a SS-free line would be phagocytized in the same way as endogenous symbionts, we injected *Ri* bacteria from the YR2(Ri) line into LL01 and YR2-Amp aphids. 24 h and 48 h post-injection, the hemolymph contained numerous free bacteria showing signs of division. *Ri* cells were observed inside adherent hemocytes, using DNA staining or specific FISH (Fig. 9C, 9E), and phagocytosis was confirmed by TEM (plasmatocytes, Fig. 9F; granulocytes Fig. 9G). We could observe phagocytosis of individual bacteria (Fig. 9F–G) and of groups of bacteria with extracellular fluid (Fig. 9F), as well as structures resembling phagolysosomes and containing *Ri* (Fig. 9G, insert). Some of the *Ri* inside phagolysosomes of granulocytes or large phagosomes of plasmatocytes showed more or less advanced signs of degradation, notably an increase in electron-density (Fig. 9F–G). Besides, hemocytes contained vacuoles of variable size as well as numerous membrane ruffles, suggesting strong endocytosis and micropinocytosis activity (Fig. 9F–G).

#### Phagocytosis of the primary symbiont *B. aphidicola*

*Buchnera* bacteria can easily be identified by light microscopy (Fig. 1A–B, 3B–D, 4A) and TEM (Fig. 2E, 7I, 8E–F) as strongly basophilic cells (intense blue coloration with MGG, Fig. 3D) with a characteristic spherical shape (2–4 µm of diameter). They were observed in hemolymph samples whatever the aphid line or the collection procedure. Although a few *Buchnera* may naturally be present in the hemolymph, they are likely released by the rupture of the bacteriocytes upon ventral dissection in most of our experiments. A variable number of *Buchnera* were observed on AHPs, some stuck on coverslips, but most of them at the surface or inside adherent hemocytes (detection by DNA staining in LL01 and YR2-Amp (Fig. 9A–B), by FISH in LL01 as an example (Fig. 9D)). Unfortunately, as most hemolymph preparations contained a low number of *Buchnera*, we were not able to obtain TEM pictures of their internalization.

## Discussion

Recent papers suggest that the aphid immune defense is surprisingly low [7], [8]. However, our knowledge remains incomplete and, for instance, the nature and functions of hemocytes were largely uncharacterized. Moreover, this lack of data also precluded addressing the question of aphid hemocytes' interaction with primary and secondary symbionts. Our study was then set up to extensively characterize immune cellular components in the pea aphid, and to document the interaction between hemocytes and symbionts in this insect model.

### Characterization of aphid circulating cells

Prior studies of aphids' hemocytes are scarce, and mainly based on light microscopy [23], [28]–[33]. The only study on *A. pisum* [24] is recent and describes three cell categories, prohemocytes, granulocytes and oenocytoids, and part of their functions. Based on morphological, histological, ultrastructural and functional criteria, we have characterized five main cell types from *A. pisum* hemolymph: prohemocytes, granulocytes, and the non previously described plasmatocytes, as well as two additional categories, spherulocytes and wax cells. Although they may not be “true” circulating cells, these last types were included since they are involved in aphid defense. At last, non-adhering cells resembling the previously described oenocytoids [24] were observed in a few hemolymph samples. As they were rare and highly labile, we could not characterize them further. Although cell characterization was performed with LL01 aphids, similar types were observed in all lines, whatever their genotype and the secondary symbiont they host.

*Wax cells* were described in the hemolymph of other aphid species, and they may have the same embryonic origin as circulating hemocytes [28]. However, they mainly localize at the base and inside the cornicles and are thus likely released in hemolymph upon collection. These cells produce the wax that sticks external aggressors [34], and their large lipid vesicle likely contains alarm pheromones emitted by attacked aphids [35].

*Spherulocytes* are mainly located under the aphid cuticle, but they were always observed in hemolymph preparations. Their location suggests they may be involved in lipid synthesis and storage, as oenocytes and oenocytoids of other insect species [36], or in providing materials for cuticle renewal. In *A pisum*, we show that spherulocytes are the main cells involved in the clotting reaction, having the same function as coagulocytes in other species [37], [38]. Spherules are also strongly reminiscent of the lipid-containing particles described as pro-coagulants in other arthropods (review in [39]), and may contain part of the coagulum-forming compounds. Upon collection, cytoplasmic spherules are quickly released and rapidly disintegrate to form fibrillar “fishnets” with the potential to sequester pathogens. Since spherulocytes are non-adherent, their previous observation as groups of cells at the surface of *Aphidius ervi* parasitoid eggs [25] may correspond to attachment of a coagulum containing entrapped spherulocytes.

Among true circulating hemocytes, prohemocytes are likely progenitor cells, able to differentiate into other hemocyte categories. Plasmatocytes and granulocytes morphologically differ from prohemocytes by the nucleus shape and the number of cytoplasmic organelles, granulocytes being recognizable from plasmatocytes by the presence of cytoplasmic granules and a larger size distribution (see AHP digital analysis, Methods S1, Fig. S2). Granulocytes and plasmatocytes also differ functionally from prohemocytes in their capacity to adhere and spread, and to phagocytize foreign bodies. Whether they correspond to different lineages or to distinct maturation stages remains to be established; characterization of cell markers will help solving this question as well as locating the yet unidentified hematopoietic organ in aphids.

### Estimation of the adherent hemocyte number

Our hemocyte counting was performed on adherent hemocytes using an automatic method. On average, 0.4 µl of hemolymph was collected per aphid and the hemocyte content was estimated at about 1440 adherent hemocytes/µl in the LL01 line. This is close to the estimate by Laughton *et al.* [24] of 1800 total hemocytes/µl, suggesting that granulocytes and plasmatocytes represent the majority of the circulating hemocytes. In comparison, L3 *Drosophila* larvae contain about 2000 to 3000 hemocytes/µl [40]. While the number of granulocytes was similar in the two aphid studies, Laughton *et al.* [24] described prohemocytes as the most abundant cells in hemolymph, which is very unlikely. Indeed, our observations suggest that the prohemocyte proportion ranges from 3% to 5%, and they are considered to be rare in insect species [23], [38], [41]. This discrepancy is certainly due to the smear technique used in [24], plasmatocytes differing mainly from prohemocytes by their adhesion capacity.

### The role of hemocytes in aphid defense

#### “Encapsulation” and melanisation

Although encapsulation of parasitoids eggs has seldom been reported in aphids, it seems to involve granule-containing cells [42] that likely correspond to granulocytes. Accordingly, *A. pisum* granulocytes, but also plasmatocytes, adhered *in vivo* to inserted foreign objects in a rather similar way to hemocytes of most insect species [41], [43]. However, the encapsulation reaction was never complete, and the “patchy” cell monolayer observed clearly differed from the well-organized capsules built by Diptera and Lepidoptera [43]. The incompleteness of the encapsulation may result from a low number of recruited hemocytes and/or a low speed of recruitment in *A. pisum*. Accordingly, the number of cells on the foreign object was much higher seven days post-insertion than after 48 h, a time at which complete encapsulation is achieved in *Drosophila*. Encystment of the “encapsulated” object in aphid tissues was often observed, which may explain the “disappearance” of parasitoid eggs in resistant strains [44].

As reported in numerous insects including other Hemiptera [3], [45], a deposit resembling melanin was observed at the surface of the foreign object. This layer might also correspond to extracellular matrix materiel deposition as suggested in Drosophila [46]. We also noticed numerous cells undergoing lysis, possibly in relation with the release of melanization enzymes such as the PO. PO activity has been described in almost all insect hemocyte types [41]; in *A. pisum*, we observed PO-positive granulocyte-type cells, as well as circulating granulocytes containing melanized latex beads in the aphid hemolymph. These cells are thus likely involved both in melanization and in the first steps of the “encapsulation” response.

### Phagocytosis of pathogenic bacteria

Plasmatocytes and granulocytes were able to massively and rapidly internalize latex beads or *E. coli* cells that had been injected into the hemolymph. Although no PGRP-encoding genes have been identified in the pea aphid genome, several genes are predicted to encode proteins involved in opsonization, which can lead to phagocytosis. They include for instance, the gram-negative bacteria binding protein 1 (GNBP1) and C-type lectins, LPS-binding proteins [7]. Dscam, the immunoglobulin superfamily receptor Down syndrome cell adhesion molecule, which may act both as a signaling receptor and an opsonin, is also present in the genome. Moreover, numerous genes acting in the phagosomal and lysosomal pathways have been identified (see KEGG pathways), as well as genes encoding surface receptors involved in triggering these pathways (e.g. integrins, multiple EGF-like-domains receptors, low-density lipoprotein receptor, CD36 like protein). This is in agreement with our observation that aphid plasmatocytes and granulocytes display the full repertoire of phagocytosis types. Finally, we observed evident signs of degradation of *E. coli* bacteria in phagolysosome-like structures, showing that aphids can destroy bacterial invaders.

Although aphid hemocytes are clearly able to perform classical immune cellular functions, it remains to be determined whether they play a significant role in aphid defense, notably against bacteria. Aphids have a limited capacity to seal wounds by coagulation and melanization [8] and were also reported to be susceptible to infection by various strains of bacteria and resistant to others [47], [48]. The efficiency of phagocytosis in controlling bacterial infections will have to be tested using various concentrations of injected or ingested bacteria, as well as with naturally occurring pathogens.

### Aphid cellular immunity and symbiosis

#### Phagocytosis of secondary symbionts

The study of adherent hemocytes in YR2(Ri), YR2-Ss and YR2-Hd lines led to the striking observation that plasmatocytes and granulocytes from each line contained a high number of the corresponding secondary symbionts (SS) in their cytoplasm. A similar result was observed following injection of *R. insecticola* symbionts into LL01 or YR2-amp aphids. The presence of symbionts inside hemocytes has previously been reported, notably in another Hemipteran species, the triatomine bug [16]. TEM analyses confirmed the presence of the SS in phagosome-like structures resembling those containing injected *E. coli*. The pea aphid hemocytes thus have the capacity to phagocytize secondary symbionts whether naturally present or injected into the hemolymph. This phagocytic capacity depends neither on the clone genotype nor on the nature of the SS (e.g. *Regiella*, *Hamiltonella*, *Serratia*). However, quantitative differences may occur, depending on these parameters.

The outcome of phagocytized SS was rather unclear compared to that of *E. coli* bacteria since no clear pictures of degradation were observed. However, the presence of many empty phagolysosomes in YR2(Ss) could suggest a rapid degradation of uptaken *Ss* bacteria, while no such indication of degradation was found for *Ri* and *Hd*. This observation and the persistence and proliferation of *Hd* and *Ri* in *Drosophila* S2 cells [49] raises the question of their possible survival inside hemocytes. These closely-related bacteria carry active type III secretion systems, and produce putative virulence factors [50], [51], in contrast to *Ss* in which many genes involved in pathogenesis seem to be inactivated [52]. Interestingly, the type III secretion system was demonstrated to be required for cell line infection and *in vivo* maintenance of the *Sodalis*-tsetse fly symbiont [53], [54]. It is also well-known that bacterial pathogens, notably other enterobacteria, use various strategies to survive host cellular defenses, promoting their own internalization and/or interfering with the phagosomal maturation processes in phagocytic cells [55], [56]. If *Ri* and/or *Hd* were demonstrated to use such strategies, their survival inside aphid hemocytes might possibly facilitate the symbionts' colonization of host tissues (such as bacteriome sheath cells and bacteriocytes), their intraspecific or interspecific horizontal transfer, or their transmission to embryos of subsequent generations. Interestingly, signs of degradation were found for *Ri* bacteria injected into LL01 or YR2-amp aphids but not for bacteria naturally present in the YR2(Ri) line. This might result either from the presence of damaged *Ri* cells following the isolation/injection procedure, or from the fact that injected *Ri* were introduced into the hemolymph of aphids previously devoid of SS.

### Adherent hemocyte number in presence of the different secondary symbionts

The difference in the number of adherent hemocytes between LL01 and YR2-amp lines, devoid of secondary symbionts, and YR2(Ri) and YR2-Hd lines clearly demonstrates that the presence of some secondary symbionts can affect aphid cellular immunity. This raises the question of how this effect is mediated. The lower adherent cell number in presence of *Hd* or *Ri* may originate from a lower number of plasmatocytes and granulocytes *in vivo*, and/or a decrease of their adhesion properties. A lower cell number may have several possible origins, including a cost of the presence of the SS on the aphid overall fitness, as previously reported [57], [58]. Strikingly, the presence of *Ss* did not affect the adherent cell number, which suggests that this effect is symbiont-dependent. Future studies will question whether the effect of the presence of a given symbiont on aphid circulating cells also depends on parameters such as the host genotype, the bacterial strain, or the conditions of infection. Finally, it is interesting to note that a reduction in immune competence has been observed in the isopod *A. vulgare* infected by Wolbachia, this bacteria being also detected host hemocytes [13], [59].

### Phagocytosis and primary symbionts

*Buchnera* (*Ba*) cells are usually enclosed inside primary bacteriocytes, surrounded by a membrane presumably derived from the host cell (the M3 membrane), and thus protected from immune cells. However, their transmission to *A. pisum* embryos is imperfect [60], possibly because it occurs by specific exocytosis from the maternal bacteriocyte [61], which may explain our sporadic observation of *Ba* in the hemolymph. When released in high number during hemolymph collection, *Ba* cells were readily taken up by hemocytes, as were foreign bacteria or SS. Douglas *et al.* [62] reported rapid lysis of *Buchnera* injected in the hemolymph of aposymbiotic aphids, as well as their uptake and rapid subsequent elimination by S2 *Drosophila* cells [63]. Although these bacteria have drastically changed due to establishment of tight symbiosis, surface molecular patterns that allow recognition by the insect immune system, such as peptidoglycan, are still present [64]; accordingly, *Drosophila* S2 cells overexpress genes encoding AMPs when infected with *Ba* [63]. In addition to the problem of the recognition pattern of *Ba* by aphid hemocytes, our results raise several questions such as whether *Ba* uptake by aphid hemocytes occurs in natural conditions, and whether *Ba* are destroyed or may be able to survive inside phagocytic hemocytes.

### Conclusion

This work shows that aphids possess all classical components of insect cellular immunity, and provides strong bases for future studies of its role in aphid defense. It also opens new areas of research in the challenging domain of the interaction between immunity and symbiosis. Based on our observations, the aphid-symbiont model may be considered as one of the most promising to address such questions as: how symbionts are perceived by the immune system, how the immune system may control their location and proliferation, and whether symbionts may still show pathogenic traits such as the ability to hide themselves in immune cells. Here, we also demonstrate that the decrease in host immunocompetence associated with some parasitic bacteria [12], [13], [59] can also occur in a case of mutualism, thus highlighting the continuum between parasitism and mutualism. Aphid symbionts might be important players in function/homeostasis of the immune system, as demonstrated for symbionts of the tsetse fly [65]. An important feature brought by the aphid model is also the evidence that different symbionts can differentially interfere with the immunity of the same host. Finally, the presence of symbionts inside aphid hemocytes might help understand the remaining mysteries associated with transmission of aphid symbionts.

## Materials and Methods

### Biological material

*A. pisum* individuals used were offspring of parthenogenetic females from two field-originating lines, LL01 and YR2(Ri), and three artificial lines (YR2-Amp, YR2-Hd, YR2-Ss) (Table 1). LL01 is devoid of secondary symbionts (SS) while YR2(Ri) (formally named YR2) only hosts *Regiella insecticola* (formerly the U-type or PAUS). YR2-Amp was produced from YR2(Ri) by elimination of *R. insecticola* using ampicillin treatment, and is therefore artificially free of SS [66]. YR2-Hd and YR2-Ss were obtained by injecting *Hamiltonella defensa* (T-type or PABS) and *Serratia symbiotica* (R-type or PASS), respectively, into the YR2-Amp line (Simon *et al.* [58]). The four YR2 lines thus differ only in their SS composition status. All strains were maintained in cages on fava bean, at 20°C, under a 16∶8 h light/dark cycle.

To obtain synchronized aphid individuals, 10 reproductive mature females were left on a plant for 24 h. The produced progeny was a synchronized cohort of aphids. All assays were carried out on adults that began to reproduce 1–2 days prior to experimentation (14–15 days-aged in our rearing conditions).

#### Hemolymph collection and adherent hemocyte preparation (AHP)

Two protocols were used for hemolymph collection. For hemolymph-clotting tests, we proceeded as described by Fukatsu *et al.* [21]. Aphids were sterilized in 70% ethanol, rinsed in water, and attached dorsally onto a Petri dish using adhesive tape. The legs were removed, and the drops of hemolymph collected using a capillary glass pre-filled or not with fresh Schneider's insect medium (SIM, Sigma). Since the collected amounts of hemolymph (0.1 µl per aphid) and the hemocytes' number were low, and the presence of cellular debris and numerous free nuclei suggested hemocytes' degradation (Fig. S1), we set up a different collection protocol for all other experiments. Briefly, aphids were sterilized and immerged into a drop (10 µl per aphid) of SIM or Grace's Insect Cell Culture Medium (Invitrogen), on a Petri dish over ice. Following careful rupture of the ventral cuticle under a stereomicroscope, the aphid-diluted hemolymph was collected. We also estimated the quantity of undiluted hemolymph recovered per aphid with this method at about 0.4 µl, using 0.5 µl glass capillaries (five repetitions). A higher number of hemocytes could be obtained by dissecting successively several aphids in the same drop of medium. For adherent hemocyte preparations (AHPs), diluted hemolymph was transferred to glass coverslips (Carl Roth, 2 cm diameter) in a multi-well plate placed in a wet chamber. Hemocytes were allowed to settle down and adhere for one hour at room temperature (15 min for detection of ROS, see below), and coverslips were then washed twice with the hemolymph collection medium.

#### F-actin and nuclei staining

Washed AHPs were fixed for 10 min with paraformaldehyde 4% (PFA 4% (Sigma) in 0.1 M phosphate buffer, pH 7.4), and washed three times, 5 min each, in phosphate-buffered saline (PBS, Sigma). To stain F-actin, AHPs were permeabilized 10 min with PBS-0.1% triton ×100, washed in PBS, and incubated one hour in the dark with Phalloidin-×5-FluoProbe 505 (Interchim; green fluorescence: Em = 530 nm) diluted 1∶200 in PBS. After PBS washing, nuclei were counterstained in the dark 10 min with 1 µg/ml of 4′,6′-diamino-2-phenylindole (DAPI, Sigma; blue fluorescence: Em = 461 nm). Next, AHPs were washed three times in PBS and one time in deionized water. Coverslips were mounted on slides using aqueous mounting medium containing an anti-fading reagent (Roti®-Mount Fluorcare, Carl Roth) and observed under a fluorescent microscope.

#### Detection of intracellular reactive oxygen species (ROS)

AHPs were incubated in the dark with non-fluorescent dihydrorhodamine 123 (DHR 123, 50 ng/ml; Sigma) diluted in SIM for 15 min RT. They were then washed once and the generation of fluorescent rhodamine 123 (resulting from intracellular oxidization of DHR 123 by ROS) was directly observed under an epifluorescent microscope with DIC filter for green fluorescence detection (Em = 534 nm).

#### Hemocyte phenoloxidase (PO) activity

PO activity in hemocytes was detected according to Ling *et al.* [67], by incubating AHPs for 30 min in freshly prepared dopamine hydrochloride (1 mg/ml in 35% ethanol, Sigma). Reaction specificity was tested using the following controls: 35% ethanol alone, and Dopamine with phenylthiourea (PTU, 2 mg/ml) that specifically inhibits PO activity. AHPs were then washed three times in PBS and coverslips were mounted on slides (Faramount Aqueous Mounting medium, Dakocytomation). PO activity was detected by the brown-dark coloration. The proportion of adherent hemocytes having PO activity was estimated by triplicate counting experiments on AHPs (five aphids dissected in 50 µl SIM per AHP).

#### May-Grünwald Giemsa (MGG) staining

AHPs were fixed with 1 ml of May-Grünwald solution (Carl Roth) for 3 min, and 1 ml of Sorensen's phosphate buffer (33.9 mM KH_2_PO_4_; 32.8 mM Na_2_HPO_4_, 12 H_2_O; pH 6.8) was then gently added for a 5 min incubation period. Coverslips were transferred in Giemsa solution (Carl Roth) 1∶20 in Sorensen's buffer for 15 min, washed in Sorensen's buffer, mounted and observed by light microscopy.

#### Nile blue sulfate staining (lipid detection)

The technique used derives from the Nile Blue method for detecting phospholipids, which stains neutral lipids pink while lipids with acidic function are stained blue [68]. Whole adult aphids were fixed with PFA 4% during one week at 4°C. After washing in PBS, they were transferred into a saturated Nile Blue A sulfate (Carl Roth) aqueous solution, and incubated at 60°C for 24 h. Aphids were then washed twice 2 h in PBS and 1 h in distilled water. Finally, they were transferred into a drop of distilled water and carefully dissected. Staining was observed by light microscopy.

#### Hemolymph clotting

The first steps of hemolymph clotting were observed using freshly collected hemolymph. 15–20 µl drops of diluted hemolymph (1∶10 in SIM) were spotted on glass slides (Carl Roth), covered with coverslips (2 cm diameter), and changes in hemocytes/hemolymph appearance were followed during 30 min by light microscopy. Alternatively, undiluted hemolymph collected from 50 aphids was spotted on glass slides. After a 1 hour in a wet chamber, 10 µl of neutral red (1 mg/ml in SIM) were gently added to the spotted drops, coverslips mounted, and the coagulum observed by light microscopy.

#### *In vivo* hemocyte adhesion assay

Aphids (LL01 line) were immobilized, the dorsal cuticle was punctured with the tip of a glass needle and a brush horsehair fragment (about 500 µm long and 80–150 µm diameter) was inserted into the hemocoele. Aphids were then returned to plants under normal rearing conditions. Fragments were removed 24 h, 48 h or 7 days after insertion, by dissecting aphids in SIM. After fixation (PFA 4%, 2 h, 4°C), washing in PBS, and staining of F-actin and DNA, fragments were examined for the presence of adhesive hemocytes. Five to seven brush horsehair fragments were observed for each time point. In addition, 20 fragments collected 7 days post-insertion were treated for TEM. To determine if other foreign objects could be “encapsulated” as well, we tested insertion of small parts of stretched glass capillary and small fragments of resin in the same manner.

#### *In vivo* phagocytosis assays

Hemocytes' phagocytic properties were tested *in vivo* by microinjecting fluorescent latex beads, fluorescent living bacteria or isolated secondary symbionts into adult aphids (LL01 and YR2-Amp). Alternatively, we looked for natural phagocytosis of SS by hemocytes of the YR2 lines using fluorescent microscopy and FISH experiments (YR2(Ri) line), and TEM (YR2(Ri), YR2-Ss and YR2-Hd). Microinjections were performed with a Nano-injector (Nanoject, Drummond) on immobilized aphids. A volume of 69 nl of sample was injected into the body cavity through the dorsal cuticle of each aphid. Injected aphids were then returned to rearing conditions until hemolymph collection.

Fluorescent latex beads (2 µm-diameter carboxylate-modified polystyrene latex beads, red fluorescent, Sigma) were washed twice with SIM. Approximately 2000 beads (adjusted after counting on a Thoma chamber) were injected *per* aphid. *E. coli* DH5-alpha expressing the red fluorescent protein (DsRed) were cultured in LB medium (Mo Bio Laboratories) and diluted in SIM. *R. insecticola* samples were obtained from YR2(Ri) aphid hemolymph. Following 5 min centrifugation at 200 g, the pellet (containing mainly embryos and bacteriocytes) was discarded, and the supernatant centrifuged at 2000 g for 5 min. The final pellet (that contained mainly SS) was then re-suspended into an adequate volume of SIM and approximately 1000 bacteria were injected *per* aphid. For “latex beads” and “fluorescent *E. coli*”, hemolymph was collected 2 h, 5 h or 24 h post-injection from pools of 10 aphids, and either directly observed or used for F-actin staining on AHPs preparation. The percentage of hemocytes phagocytizing fluorescent latex beads was estimated by counting on AHPs the number of adherent hemocytes containing three or more beads, 5 h (1029 hemocytes analyzed) and 24 h (1023 hemocytes analyzed) post-injection. For TEM, the hemolymph was collected 24 h post-injection (see below). For *“R. insecticola”* injection, DNA staining and FISH detection on AHPs were performed with hemolymph collected 24 h and 48 h post-injection, while TEM samples were prepared from a collection 24 h post-injection.

#### Estimation of adherent hemocytes' number

The total number of hemocytes in hemolymph samples could not be directly determined (see results), but we nevertheless estimated the number and proportions of the different types of adherent hemocytes, i.e. plasmatocytes and granulocytes. F-actin stained AHPs from five aphids were photographed under a fluorescent microscope using a video camera, and the digital images were processed and analyzed using a specific routine developed using the ImageJ 1.42q software (http://rsbweb.nih.gov/ij/; for details see Methods S1 and Fig. S2). Estimations were made in four replicates for each aphid line. Results were analyzed with the one-way ANOVA and Tukey multiple comparisons of means (95% family-wise confidence level) test (TukeyHSD), after verification of assumptions (normality and homogeneity of variances), using the R 2.12.1 software (www.r-project.org/).

#### Fluorescent in situ hybridization (FISH)

FISH was performed according to Tsuchida *et al*. [69] on AHPs fixed for 10 min in 4% paraformaldehyde. The following probes were used for detection of symbionts' 16S rRNA: ApisP2a-Cy3 (5′-Cy3-CCTCTTTTGGGTAGATCC-3′) for *B. aphidicola*, and U16-Cy5 (5′-Cy5-GTAGCAAGC TACTCCCCGAT-3′) for *R. insecticola* (U type). Briefly, hybridization buffer (20 mM Tris-HCl, pH 8.0; 0.9 M NaCl; 0.01% sodium dodecyl sulfate; 30% formamide) containing 10 pmol/ml of probe and 200 ng/ml of DAPI was applied on fixed AHPs. Slides were incubated in a wet chamber at 46°C overnight, rinsed 10 min in 1× SSC (0.15 M NaCl, 15 mM sodium citrate) and 1 min in deionized water, mounted with fluorescent mount medium and observed under epifluorescent or confocal microscope to detect *B. aphidicola* and *R. insecticola* (red fluorescence and far-red fluorescence respectively). No-probe experiments were performed as a control.

#### Transmission electron microscopy (TEM)

TEM sample blocks were prepared from the pooled hemolymph of 100–200 aphids. Approximately ten samples of diluted hemolymph, each from 20 aphids (dissected into 200 µl of SIM), were pooled into a centrifuge vial, on ice. The same volume of fixative (4% glutaraldehyde (Sigma) in 0.1 M sodium cacodylate buffer, pH 7.2) was added to the vial that was next conserved 24 h at 4°C. Fixed hemolymph was then centrifuged (500 g, 10 min) to pellet hemocytes and remove the fixative. The pellet was washed and diluted in the same buffer, filtered through a 48 µm filter to remove embryos and tissues debris, and centrifuged again (500 g, 10 min). Post-fixation was done by 2% osmium tetroxide in cacodylate buffer. Following dehydration in graded series of ethanol solutions, samples were embedded into Epon resin. The brush horsehair fragments removed from aphid hemocoele 7 days after insertion (see above) were put into fixative (2% glutaraldehyde in 0.1 M sodium cacodylate buffer, pH 7.2) for 24 h at 4°C. After washing in the same buffer, the brush horsehair fragments were post-fixed and embedded as described above. Samples sections were cut with a diamond knife using a LKB ultramicrotome, mounted on copper grids, stained with uranyl acetate and lead citrate, and observed with a Zeiss EM10CR electron microscope at 80 kV.

#### Light and Fluorescent Microscopy

Samples were examined using epifluorescent microscopes or a confocal microscope, all fitted with differential interference contrast (DIC) optics. The microscope “Axioplan 2” (ZEISS) with the objective “Plan Neofluar 40×/0.75” and the color camera “Axiocam color” was used for acquisition of colored images without fluorescent staining. The microscope “Imager.Z1” (ZEISS) with objectives “EC Plan-Neofluar 40×/0.75” and “Plan Apochromat 63×/1.4 oil” and the black and white camera “Axiocam MRm” was used for acquisition of epifluorescent images and for black and white DIC images. All confocal micrographs were acquired with the microscope “LSM 510 Meta” (ZEISS) coupled with the ZEISS software and using the objective “Plan-Neofluar 40×/1.3 oil”. All captured images were exported to Adobe Photoshop for figure assembly.

## Supporting Information

Figure S1 ***In vivo*** **observation of free nuclei originating from lysed spherulocytes.** (**A**) Spherulocyte losing its spherules 5 min after hemolymph collection. The nucleus and the central highly stained blue nucleolus are visible. (**B**) Nucleus from a lysed spherulocyte, 20 min after hemolymph collection. The nucleus is associated with a few spherules and cellular debris. (**C–E**) Free spherulocyte nucleus, 30 min after hemolymph collection. (**C**) Methyl blue staining. (**D**) DNA staining using DAPI. **E.** Merge of **C** and **D.** Legends: arrow: nucleus; arrowhead: nucleolus. Scale bar: 10 µm.
(TIF)

Figure S2 **Estimation of adherent hemocyte numbers by image processing and analysis.** (**A**) Representative examples of visually classified thumbnails of F-actin stained adherent particles into four subgroups: undefined particles, plasmatocytes, granulocytes and clusters of cells. (**B**) Outlines of the particles presented in A after image processing and particle analysis using ImageJ. (**C**) Plots of two measurements recorded by ImageJ on the 244 visually pre-classified thumbnails. The Feret's diameter measurement did not accurately discriminate the pre-established groups in contrast to the Area's measurement. Dotted lines represent the threshold values of the hemocyte Area that allows distinguishing plasmatocytes (40 µm^2^<Area<250 µm^2^) from granulocytes (250 µm^2^<Area<1500 µm^2^). Undefined particles (Area<40 µm2) and part of cell clusters (Area>1500 µm2) were removed from the analysis. Scale bars: 20 µm for **A** and **B**.
(TIF)

Methods S1 **Adherent hemocyte counting.**
(DOCX)

Movie S1 **Observation of the clotting process.** The movie first shows a degraded spherulocyte surrounded with numerous fibrils of small size. Successive steps of the clotting process are then observed, with isolated cytoplasmic blebs undergoing vacuolization, and formation of long strands like strings of pearls.
(AVI)

Movie S2 **3D reconstitution of F-actin stained hemocytes partially covering a fragment of horsehair 7 days after insertion in the body cavity of an LL01 adult aphid.** The horsehair is the same as in Fig. 6A. 3D-reconstitution was generated from 20 confocal optical sections.
(MP4)

Movie S3 **3D reconstitution generated from confocal optical sections of an adherent granulocyte having engulfed numerous latex-beads.** F-actin: green fluorescence; latex-beads: red fluorescence.
(MP4)
