# Supplementary figures and images for: The Cellular Immune Response of the Pea Aphid to Foreign Intrusion and Symbiotic Challenge
Source: PLoS One. 2012 Jul 27;7(7):e42114. doi: 10.1371/journal.pone.0042114 (PMC3407134; doi:10.1371/journal.pone.0042114)

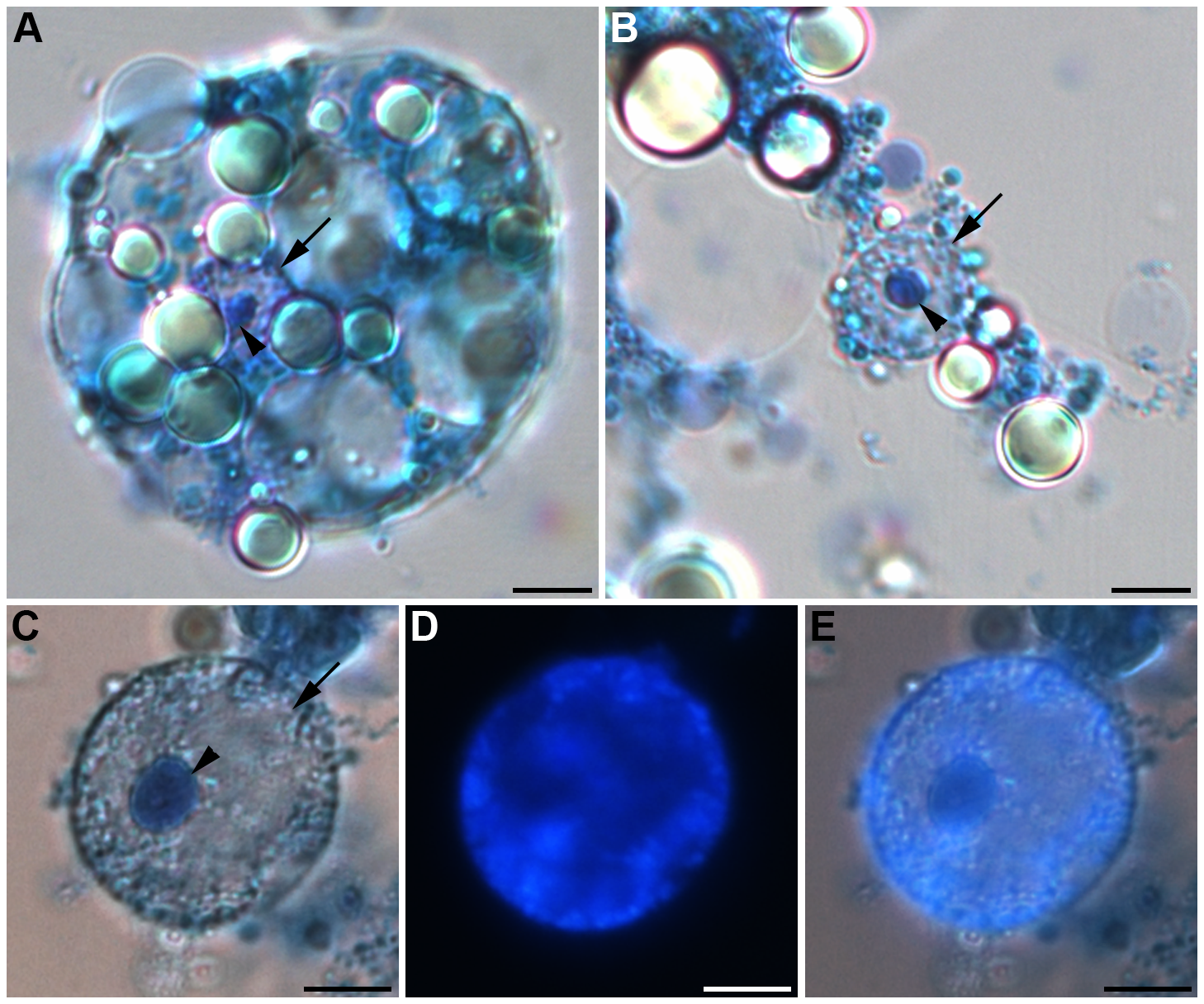

Supplement: Figure S1 — In vivo observation of free nuclei originating from lysed spherulocytes. (A) Spherulocyte losing its spherules 5 min after hemolymph collection. The nucleus and the central highly stained blue nucleolus are visible. (B) Nucleus from a lysed spherulocyte, 20 min after hemolymph collection. The nucleus is associated with a few spherules and cellular debris. (C–E) Free spherulocyte nucleus, 30 min after hemolymph collection. (C) Methyl blue staining. (D) DNA staining using DAPI. E. Merge of C and D. Legends: arrow: nucleus; arrowhead: nucleolus. Scale bar: 10 µm. (TIF) [file pone.0042114.s001.tif]

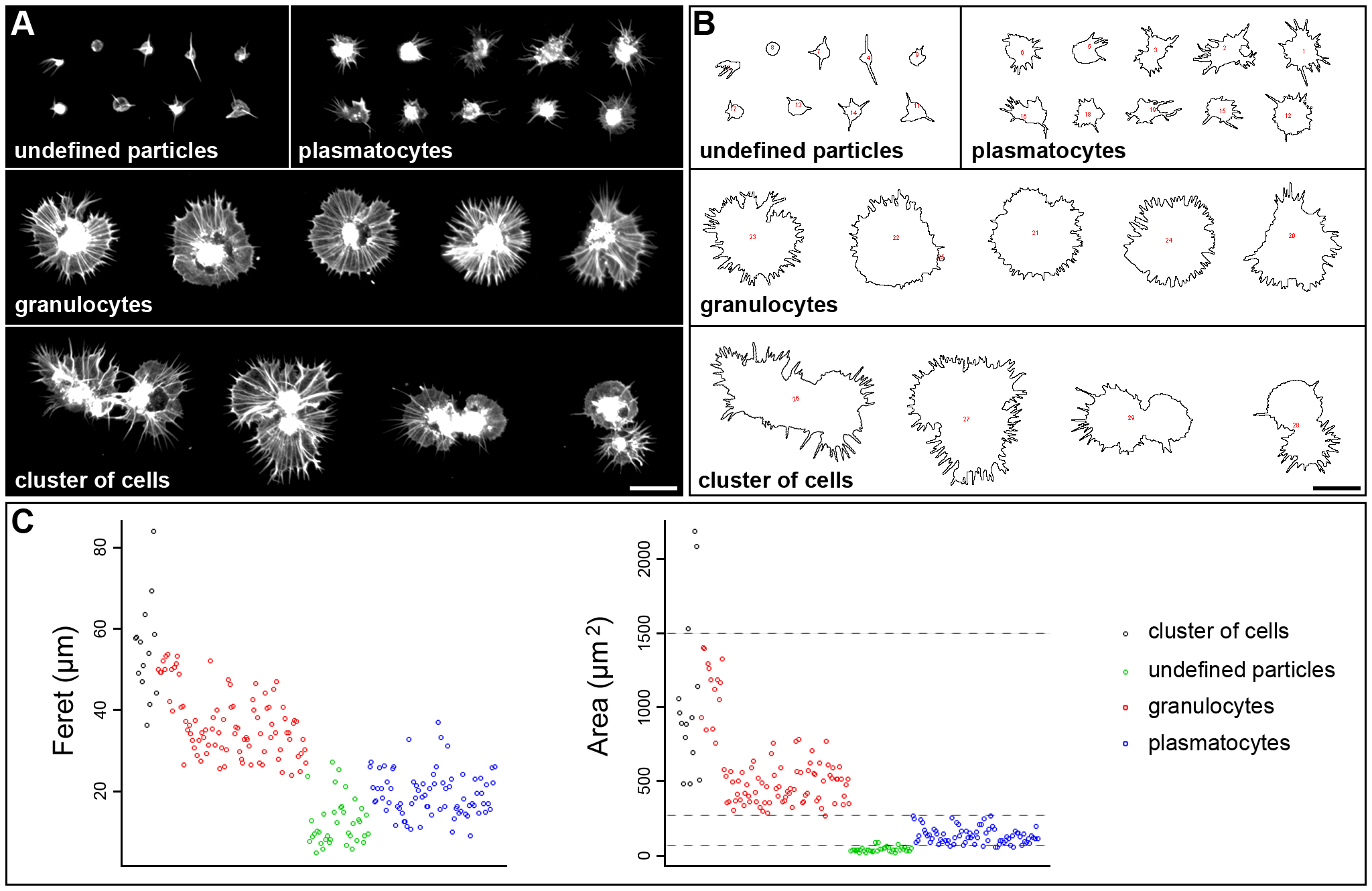

Supplement: Figure S2 — Estimation of adherent hemocyte numbers by image processing and analysis. (A) Representative examples of visually classified thumbnails of F-actin stained adherent particles into four subgroups: undefined particles, plasmatocytes, granulocytes and clusters of cells. (B) Outlines of the particles presented in A after image processing and particle analysis using ImageJ. (C) Plots of two measurements recorded by ImageJ on the 244 visually pre-classified thumbnails. The Feret's diameter measurement did not accurately discriminate the pre-established groups in contrast to the Area's measurement. Dotted lines represent the threshold values of the hemocyte Area that allows distinguishing plasmatocytes (40 µm2<Area<250 µm2) from granulocytes (250 µm2<Area<1500 µm2). Undefined particles (Area<40 µm2) and part of cell clusters (Area>1500 µm2) were removed from the analysis. Scale bars: 20 µm for A and B. (TIF) [file pone.0042114.s002.tif]
