# Supplementary material for: The Cellular Immune Response of the Pea Aphid to Foreign Intrusion and Symbiotic Challenge
Source: PLoS One. 2012 Jul 27;7(7):e42114. doi: 10.1371/journal.pone.0042114 (PMC3407134; doi:10.1371/journal.pone.0042114)
Supplement: Methods S1 — Adherent hemocyte counting. (DOCX) [file pone.0042114.s003.docx]

**Methods S1. Adherent hemocyte counting.**

*Estimation of the parameters for hemocyte discrimination.* First, 244 thumbnails of F-actin stained particles from microscopic acquisitions (8-bit black and white images) were visually classified into four groups: undefined particles, plasmatocytes, granulocytes, and cell clusters (Fig. S2A for representative examples). Thumbnails were thresholded to obtain binary images usable for subsequent analysis. Particles analysis was then processed on these pre-classified thumbnails using measurements basically implemented in ImageJ: *Area*, *Perimeter*, *Fit Ellipse,* *Feret’s diameter* and *Shape Descriptors* (see a detailed description at http://rsb.info.nih.gov/ij/docs/menus/analyze.html#set ; see outlines of the particles shown Fig. 4A 2A, after ImageJ treatment, Fig. 4B2B). The plots of the recorded measurements (distribution of the 244 particles analyzed for each type of measurement and for each defined group) allowed to (i) choose the *Area* as the measurement allowing the best discrimination between groups and (ii) determine limit values of this parameter for each group (Fig S2C). Undefined particles (Area <40 µm2) and part of cell clusters (Area >1500 µm2) could be discarded, plasmatocytes and granulocytes being defined as particles with 40 µm2<Area<250 µm2 and 250 µm2<Area<1500 µm2, respectively.

*Estimation of the differential hemocyte counts.* The estimation of the number and proportions of plasmatocytes and granulocytes was done in triplicate. AHPs were performed using three 14-days old synchronized aphids that were bled into 50 µl Schneider’s Insect medium, before F-actin staining was performed (as described in material and methods). Black and white images corresponding to the totality of the adherent hemocytes were acquired, all with the same exposure time, using the objective “Plan-Neofluar 10X/0.3”of an Imager Z1 microscope (Zeiss). Approximately 100 images were acquired per AHP at this magnification. Raw images were then exported as tiff images, threshold values were determined manually and images were automatically processed and analyzed by a batch process. The ImageJ macro used for automatical hemocyte counts was as follows:

{ repertoire=getDirectory("Choose a Directory"); list=getFileList(repertoire);

getFileList(repertoire); for (i=0; i<list.length; i++) {open(repertoire+list[i]); traitement();}

function traitement() {run("Set Scale...", "distance=310 known=200 pixel=1 unit=µm global"); run("8-bit"); setAutoThreshold(); //run("Threshold..."); setThreshold(45, 255); run("Convert to Mask"); run("Fill Holes"); run("Set Measurements...", "area display redirect=None decimal=1"); run("Analyze Particles...", "size=40-1500 circularity=0.00-1.00 show=Outlines display exclude"); close(); close();} save(repertoire+"results.txt");}

Threshold and scale values depend on the exposure time and image size respectively, and had to be determined manually.
